# Effect of catheter ablation with vein of Marshall ethanol infusion for perimitral flutter in a patient with senile transthyretin cardiac amyloidosis: a case report

**DOI:** 10.3389/fcvm.2025.1589051

**Published:** 2025-07-24

**Authors:** Simon Fitouchi, Mickael Ohana, Thomas Cardi, Laurence Jesel, Halim Marzak

**Affiliations:** ^1^ Division of Cardiovascular Medicine, Nouvel Hôpital Civil, Strasbourg University Hospital, Strasbourg, France; ^2^ Department of Radiology, Nouvel Hôpital Civil, Strasbourg University Hospital, Strasbourg, France; ^3^ UR 3074 Translational Cardiovascular Medicine CRBS, Strasbourg, France

**Keywords:** bipolar voltage map, vein of Marshall, cardiac amyloidosis, catheter ablation, atrial fibrillation

## Abstract

**Background:**

Senile transthyretin cardiac amyloidosis (AL) is an underdiagnosed infiltrative cardiomyopathy causing heart failure symptoms in elderly patients. It is associated with a higher incidence of atrial fibrillation (AF) and atrial flutter.

**Case summary:**

A 75-year-old male patient with senile transthyretin cardiac AL presented with congestive heart failure [New York Heart Association (NYHA) IV] related to rapid perimitral atrial flutter, causing tachycardia-induced cardiomyopathy with a left ventricular ejection fraction (LVEF) of 25%. He underwent AF voltage-guided ablation with vein of Marshall (VOM) ethanol infusion to block the mitral isthmus. Left atrial bipolar voltage mapping revealed diffuse and severe left atrial low-voltage areas related to amyloid protein infiltration within the left atrium (LA). After a 48-month follow-up, no arrhythmia recurrence was observed. Heart failure symptoms improved significantly (NYHA I–II) with an improved LVEF of approximately 45%–50%.

**Discussion:**

Diffuse and severe left atrial fibrosis related to amyloid protein infiltration within the LA is generally associated with worse catheter ablation outcomes in cardiac AL patients. This case demonstrated that the VOM ethanol infusion was critical to the success of the catheter ablation procedure, resulting in a better long-term outcome.

## Introduction

Wild-type transthyretin (ATTRwt) cardiac amyloidosis (AL), also known as senile cardiac amyloidosis (SCA), is an underdiagnosed form of restrictive cardiomyopathy causing heart failure in elderly patients. Men over 65 years are predominantly affected (1). The diagnosis is increasing with the aging population and the development of non-invasive diagnostic imaging technologies. ATTRwt patients present a higher incidence of atrial fibrillation (AF) and atrial flutter compared with those with ATTRm and AL cardiac amyloidosis. Atrial arrhythmia can be the first manifestation at the time of diagnosis of cardiac amyloidosis (CA) (1, 2). The arrhythmic substrate is characterized by interstitial extracellular infiltration with amyloid protein in both ventricles and atria.

## Case presentation

We report the case of a 75-year-old man referred to our institution for catheter ablation of persistent left atrial flutter. This arrhythmia had caused tachycardia-induced cardiomyopathy with a left ventricular ejection fraction (LVEF) of approximately 25% in April 2019. He experienced heart failure symptoms (NYHA IV) and had a dual-chamber pacemaker for high-degree atrioventricular block. His medical history included prostatic adenocarcinoma. The patient's medical history did not reveal any extracardiac manifestations, such as carpal tunnel syndrome, autonomic dysfunction, or lumbar canal stenosis.

Biological assessment revealed an elevated NT-proBNP level at 5,199 ng/L (normal <450 ng/L) and a normal troponin level at 11.5 ng/L (normal <57 ng/L). The electrocardiogram showed left atrial flutter at 87 bpm with a complete right bundle branch block (QRS duration of 179 ms) and a left anterior fascicular block (Supplementary Figure S1).

An external electric shock at 200 J was performed during the same hospitalization, with recurrence under amiodarone 30 days later.

**Figure 1:**
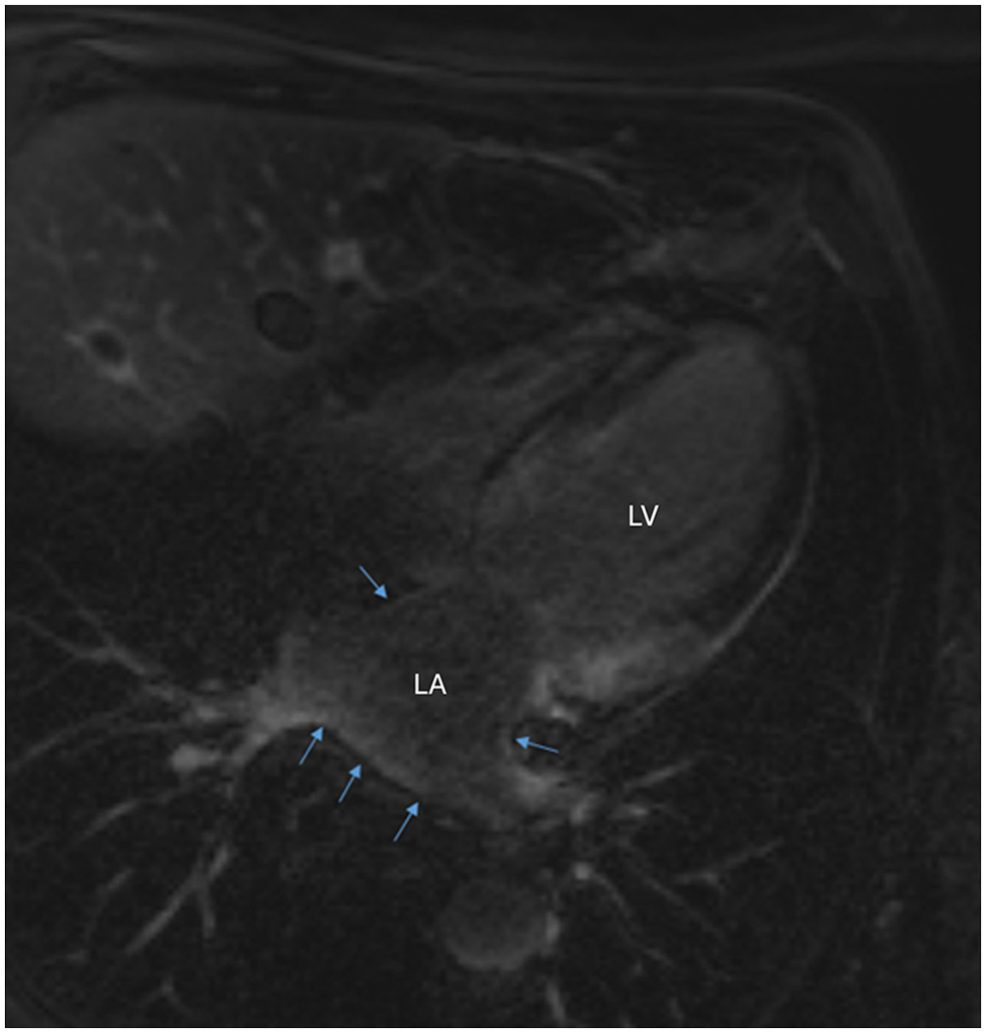
Cardiac magnetic resonance imaging demonstrating diffuse subendocardial late gadolinium enhancement in the left atrium at the level of superior pulmonary veins (blue arrows). LA, left atrium; LV, left ventricle.

Prior to the recurrence of atrial flutter, the transthoracic echocardiography demonstrated a recovery of LVEF at 35%, with a non-dilated left ventricle and diffuse hypokinesia. There was moderate left ventricular hypertrophy (posterior wall and septal thickness at 14 mm), and good right ventricular function. Cardiac magnetic resonance imaging on 9 July 2019 showed late gadolinium enhancement (LGE) extending subendocardially over both ventricles, the interatrial septum, and both atria, strongly suggesting the diagnosis of cardiac amyloidosis. Diffuse subendocardial LGE was observed in the left atrium (LA) at the level of superior pulmonary veins (Figure 1).

Technetium 99m-labeled 3,3-diphosphono-1,2-propanodicarboxylic acid (99mTc-DPD) scintigraphy showed no bone metastases but displayed significant cardiac uptake, suggesting senile transthyretin cardiac amyloidosis. The Perugini score was 2.

Protein electrophoresis and light chain assay were normal. Bence Jones protein analysis was negative, excluding the diagnosis of AL cardiac amyloidosis. The diagnosis of cardiac amyloidosis was strongly suggested by the findings on cardiac MRI and bone scintigraphy, which obviated the need for endomyocardial biopsy with Congo red staining. The most likely diagnosis was SCA in our patient. He underwent an electrophysiological study under local anesthesia, which confirmed left atrial flutter.

Three weeks later, a catheter ablation procedure with vein of Marshall (VOM) ethanol infusion was performed under general anesthesia, using a three-dimensional electroanatomic mapping system (CARTO3, Biosense Webster, Inc., CA, USA). Two transseptal punctures were made. A high-density catheter (PentaRay, Biosense Webster, Inc., CA, USA) was used to map the left atrium (LA). This catheter features 1 mm electrodes with interelectrode spacings of 2-6-2 mm. Tissue ablation was performed using a 4 mm irrigated contact-force ablation catheter (ThermoCool SmartTouch, Biosense Webster, Inc., CA, USA).

During the procedure, the activation map showed a counterclockwise perimitral macro-reentrant flutter with an atrial cycle length of 260 ms (Supplementary Video S1, Figure S2).

Entrainment maneuvers with post-pacing interval measurements were not performed because the activation map clearly indicated a perimitral flutter.

**Figure 2:**
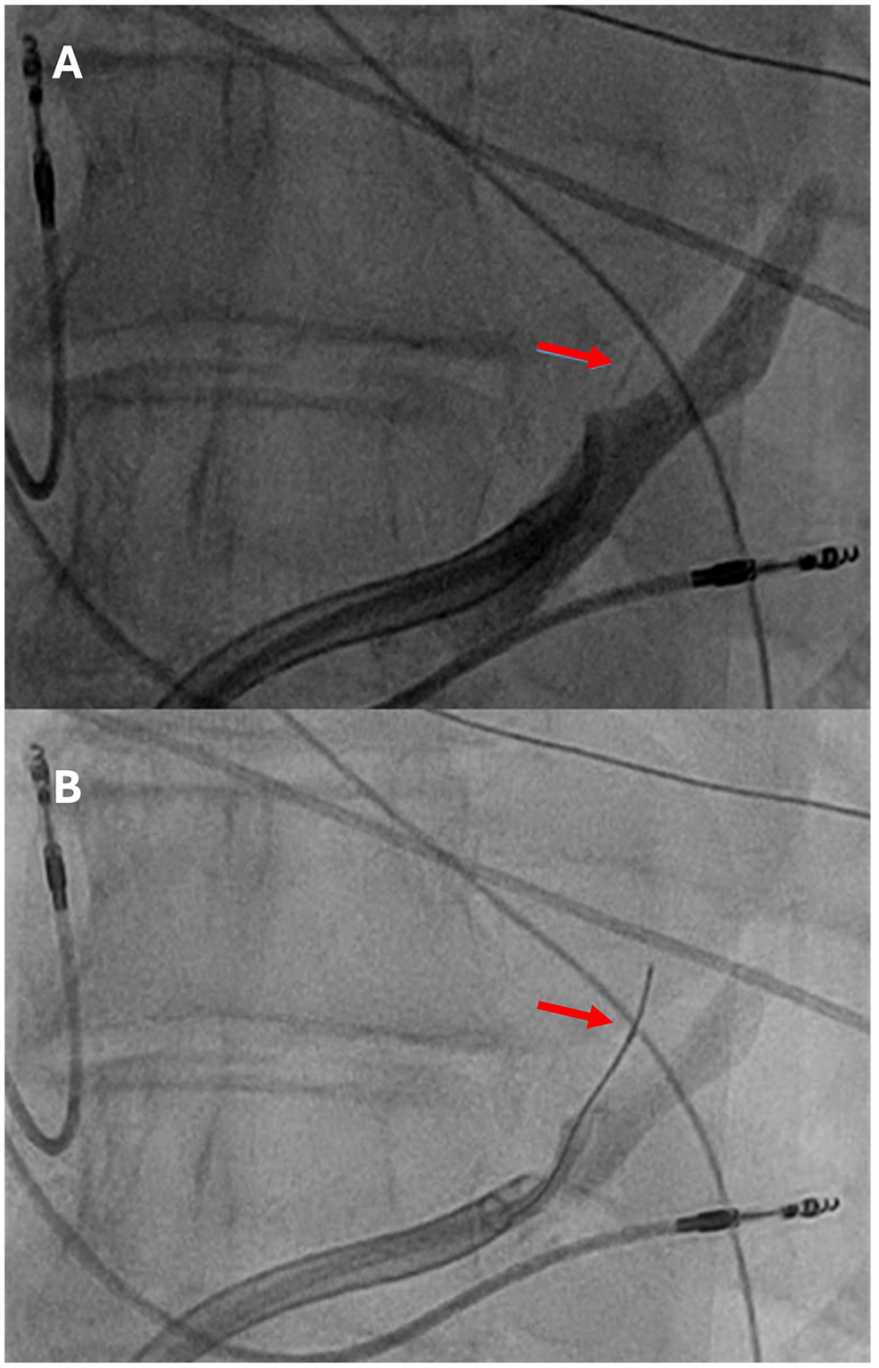
**(A)** Contrast injection identified the vein of Marshall (red arrow), which was subsequently cannulated with an angioplasty wire and a 1.5 mm  × 6 mm angioplasty balloon **(B)**.

**Figure 3:**
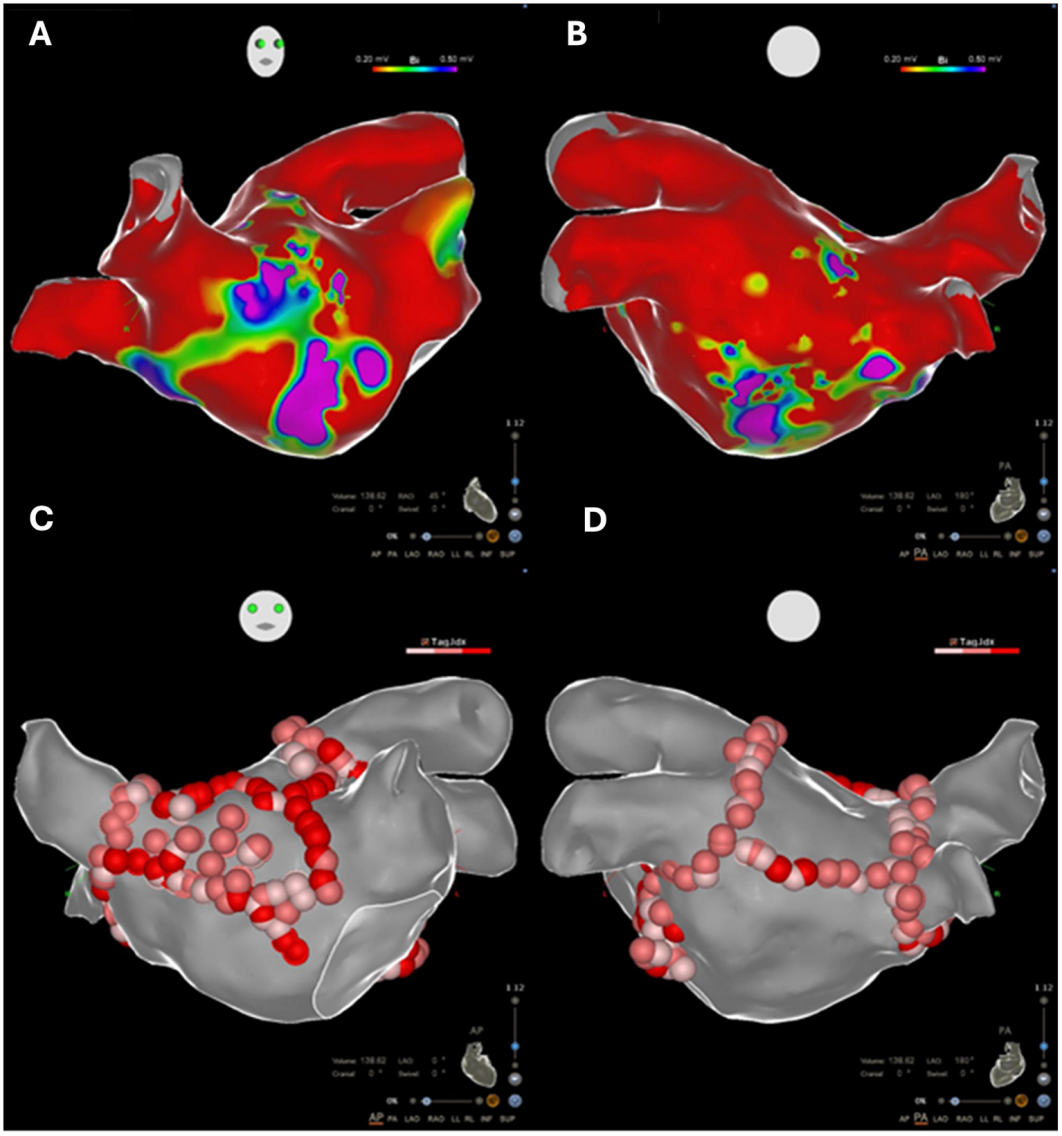
**(A,B)** Electroanatomic voltage maps demonstrating diffuse low-voltage areas in the left atrium (bipolar <0.5 mV), compatible with extensive scarring related to amyloid infiltration. **(C,D)** Linear ablative approach performed in the diffusely low-voltage left atrium.

Endocardial linear ablation of the mitral isthmus slowed the atrial flutter cycle length to 290 ms (Supplementary Figure S3). The second activation map revealed a blocked endocardial mitral isthmus with an anticlockwise passage through the VOM (Supplementary Video S2). The VOM was identified by coronary sinus (CS) angiography using a sub-selector catheter with a ∼90° angle at the tip advanced through the CS sheath. It was then cannulated with an angioplasty wire and balloon (1.5 × 6 mm) (Figures 2A,B). A total of 9 ml of ethanol was delivered to the VOM. The first ethanol injection restored sinus rhythm, achieving bidirectional mitral isthmus block. While pacing from the left atrial appendage with the high-density catheter, the activation sequence in the CS was from proximal to distal, consistent with a complete mitral isthmus line block (Supplementary Figure S4). Differential pacing was also performed from the proximal CS to the line of block, with recordings taken on the lateral side of the line. LA geometry and endocardial bipolar voltage mapping were performed in sinus rhythm using the high-density catheter. The maps displayed diffuse low voltage in the LA, compatible with extensive scarring related to amyloid infiltration (Figures 3A,B). The overall LA median bipolar voltage amplitude was 0.1 mV, with LA volume measured at 139 ml. A linear LA ablation approach was chosen due to diffuse low voltage in the left atrium. Posterior left atrial wall isolation was performed using roof and inferior lines following pulmonary vein isolation. Additionally, an anteroseptal line was created from the septal mitral annulus to the right superior pulmonary vein to avoid septal atrial flutter (Figures 3C,D), achieving bidirectional block as confirmed by pacing from the left atrial appendage with the high-density catheter.

No atrial arrhythmia was induced at the end of the procedure. During the 48-month follow-up, no arrhythmic events were observed in our patient without discontinuing amiodarone. Heart failure symptoms improved significantly (NYHA I–II). An improvement in LVEF to approximately 45%–50% was observed on transthoracic echocardiography, suggesting transthyretin amyloid cardiomyopathy with rhythmic involvement.

## Discussion

We observed diffuse low-voltage areas (bipolar <0.5 mV) in LA voltage maps during sinus rhythm in cardiac amyloidosis patients undergoing ablation, as mentioned in several studies (3–5). This amyloid protein infiltration in the left atrium is characterized by diffuse subendocardial LGE on cardiac magnetic resonance imaging. Thus, extensive left atrial disease on voltage mapping should prompt physicians to consider cardiac amyloidosis. Severe left atrial fibrosis is known to be associated with frequent arrhythmia recurrences in AF patients undergoing catheter ablation (6). Previous studies have reported poor catheter ablation outcomes in cardiac amyloidosis patients, with 1-year recurrence rates of 60% (4) and 50% (5) without VOM ethanol infusion. The ablation procedure was performed without complications in our patient, although it is known that atrial fibrillation ablation in patients with cardiac amyloidosis may be associated with an increased risk of acute clinical adverse events in the short term (notably pericardial effusion and significant hemorrhagic events) and mortality, compared with patients with heart failure without cardiac amyloidosis (6).

In our patient, no atrial arrhythmic event was observed after 48 months of follow-up, and heart failure symptoms markedly improved, with no hospitalizations for acute decompensated heart failure during follow-up. This duration of follow-up remains insufficient, given the high risk of recurrence in this population. In cases of uncontrolled heart rate despite ablation, atrioventricular node ablation, with or without pacemaker implantation, should be considered.

The success of the catheter ablation procedure was also achieved thanks to the use of ethanol infusion into the VOM, particularly in patients with a severely dilated left atrium and advanced cardiac amyloidosis. The alcoholization of the VOM represents a promising complementary approach in the management of persistent atrial fibrillation, especially in refractory cases. This technique targets arrhythmogenic epicardial tissue that is often inaccessible to conventional endocardial ablation, thereby improving the outcomes of catheter ablation in patients with cardiac amyloidosis.

## Conclusion

This case report demonstrates successful management of persistent left atrial flutter in a senile transthyretin cardiac amyloidosis patient using catheter ablation with VOM ethanol infusion. Despite the challenging substrate of diffuse low-voltage areas, the procedure achieved long-term arrhythmia control and significant improvement in heart failure symptoms. The use of VOM ethanol infusion was critical in achieving bidirectional mitral isthmus block and may have contributed to the favorable outcome. This report highlights the potential of advanced ablation techniques in managing atrial arrhythmias in CA patients, who typically have poor outcomes with conventional approaches. However, further research is needed to validate the efficacy of VOM ethanol infusion in larger cohorts and to establish optimal patient selection criteria for this approach in cardiac amyloidosis patients with refractory atrial arrhythmias.

## Data Availability

The raw data supporting the conclusions of this article will be made available by the authors, without undue reservation.

## Supplementary material

Supplementary Figure S1 Twelve-lead electrocardiogram prior to ablation showing a left atrial flutter with complete right bundle branch block and left anterior fascicular block.

Supplementary Figure S2 Intracardiac tracing demonstrating a counterclockwise perimitral macro-reentrant flutter with an atrial cycle length of 260 ms.

Supplementary Figure S3 Endocardial linear ablation of the mitral isthmus slowed the atrial flutter cycle length to 290 ms.

Supplementary Figure S4 Bidirectional mitral isthmus block confirmed by pacing from the left atrial appendage with the high-density catheter.

Supplementary Video S1 Video loop of the activation map demonstrating a counterclockwise perimitral macro-reentrant atrial flutter with an atrial cycle length of 260 ms.

Supplementary Video S2 Video loop of the activation map, after endocardial linear ablation of the mitral isthmus, demonstrating a blocked endocardial mitral isthmus with counterclockwise conduction through the vein of Marshall.
