# Supplementary figures and images for: Effect of catheter ablation with vein of Marshall ethanol infusion for perimitral flutter in a patient with senile transthyretin cardiac amyloidosis: a case report
Source: Front Cardiovasc Med. 2025 Jul 24;12:1589051. doi: 10.3389/fcvm.2025.1589051 (PMC12328312; doi:10.3389/fcvm.2025.1589051)

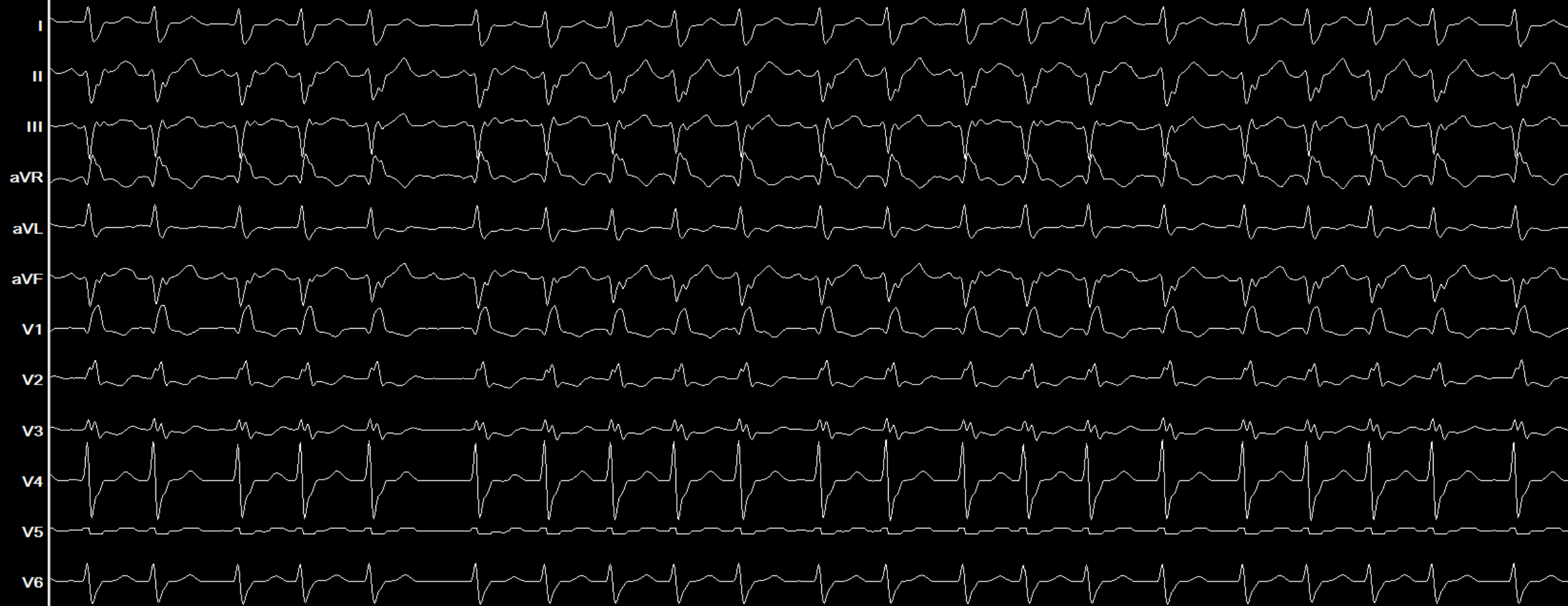

Supplement: Supplementary Figure S1 — Twelve-lead electrocardiogram prior to ablation showing a left atrial flutter with complete right bundle branch block and left anterior fascicular block. [file Datasheet1.pdf]

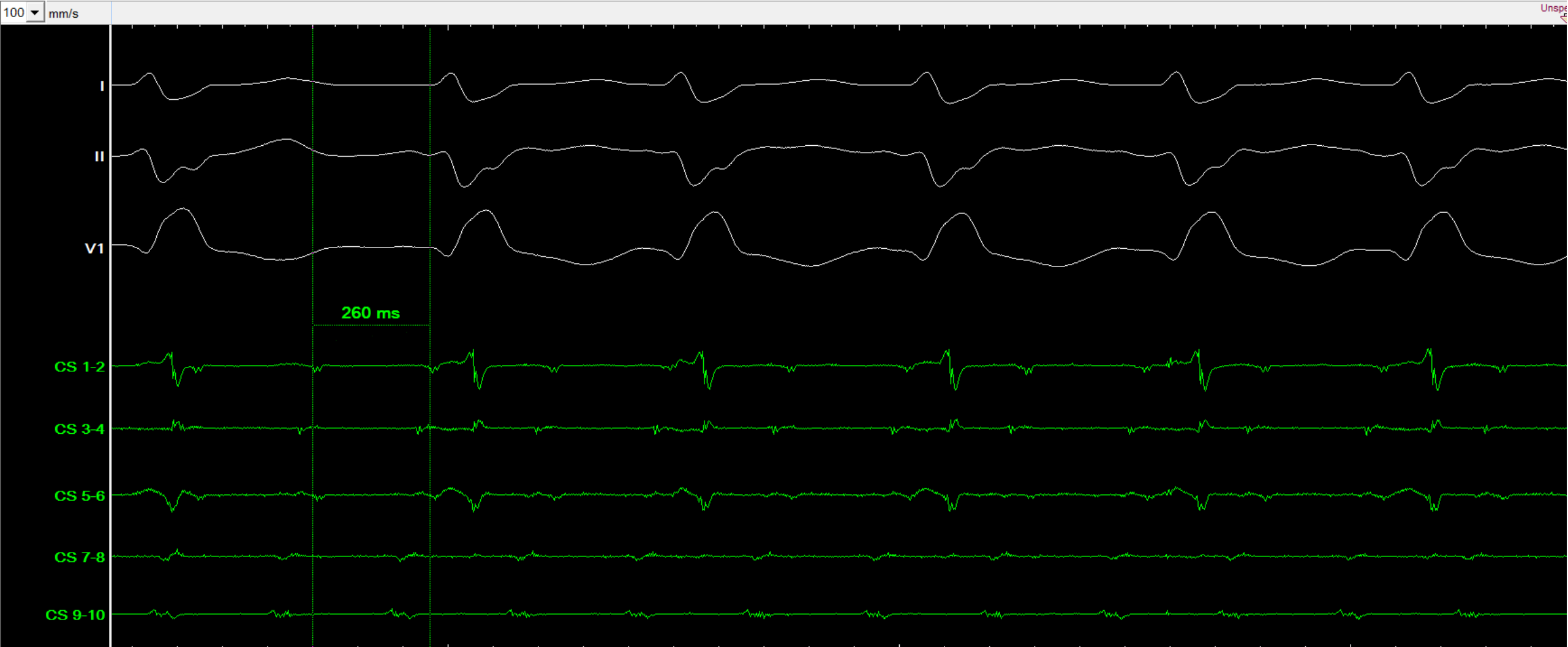

Supplement: Supplementary Figure S2 — Intracardiac tracing demonstrating a counterclockwise perimitral macro-reentrant flutter with an atrial cycle length of 260 ms. [file Datasheet2.pdf]

100 mm/s

Ablatio

I

II

V1

CS 1-2

CS 3-4

CS 5-6

CS 7-8

CS 9-10

290 ms

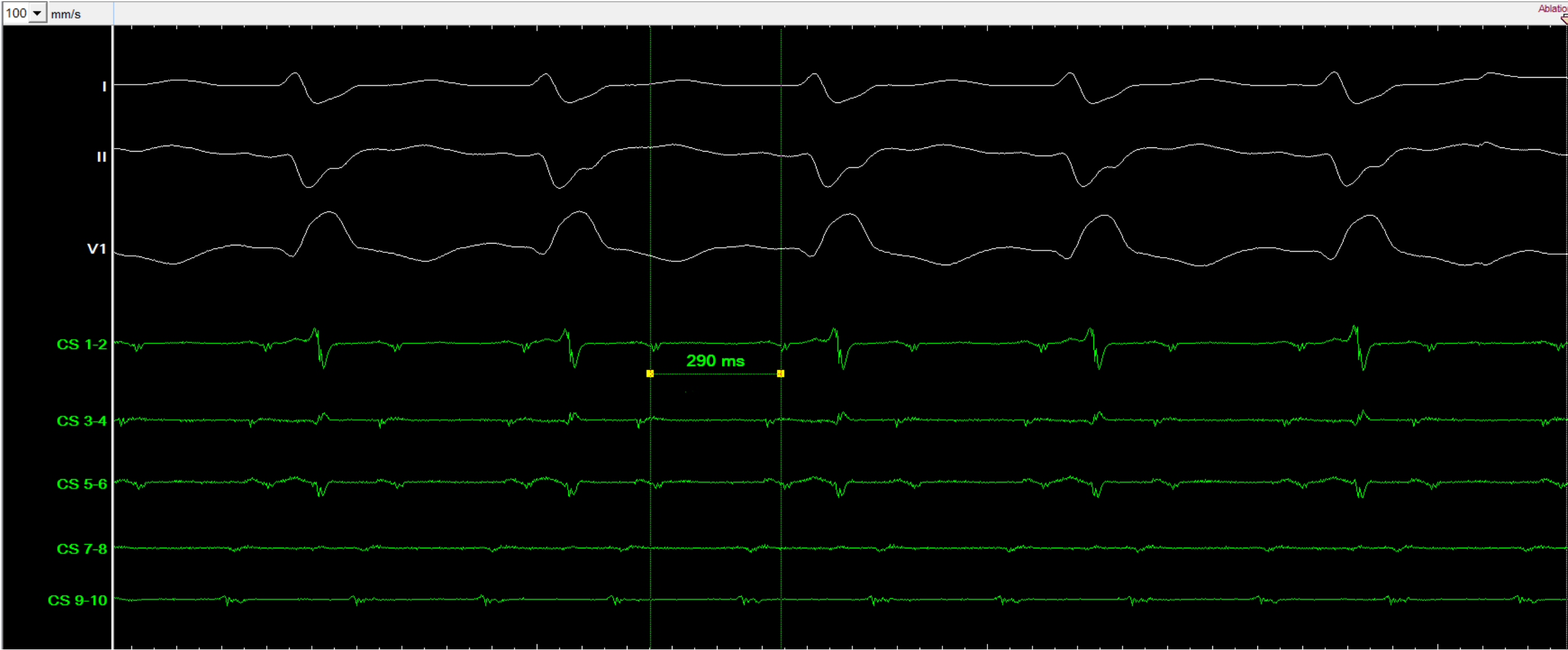

Supplement: Supplementary Figure S3 — Endocardial linear ablation of the mitral isthmus slowed the atrial flutter cycle length to 290 ms. [file Datasheet3.pdf]

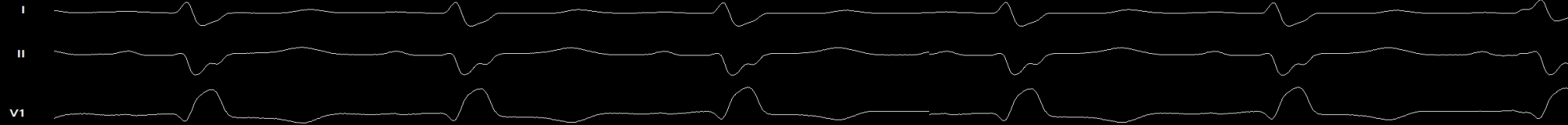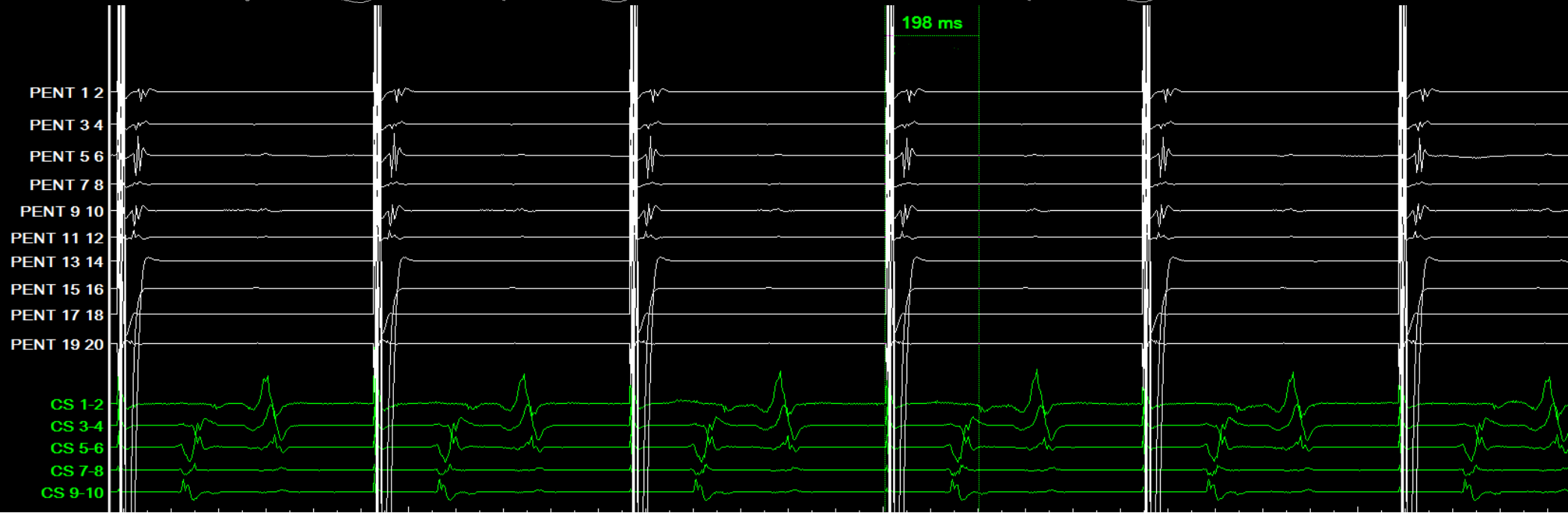

Supplement: Supplementary Figure S4 — Bidirectional mitral isthmus block confirmed by pacing from the left atrial appendage with the high-density catheter. [file Datasheet4.pdf]
